# Sarcopenic osteoporosis, sarcopenic obesity, and sarcopenic osteoporotic obesity in the Camargo cohort (Cantabria, Spain)

**DOI:** 10.1007/s11657-022-01146-1

**Published:** 2022-07-29

**Authors:** Paula Hernández-Martínez, José M. Olmos, Javier Llorca, José L. Hernández, Jesús González-Macías

**Affiliations:** 1 Hospital Sierrallana, Torrelavega, Cantabria Spain; 2 Department of Internal Medicine, Bone Metabolic Unit, Hospital Universitario Marqués de Valdecilla, Avda. Valdecilla s/n. 39008, Santander, Cantabria Spain; 3 IDIVAL (Instituto de Investigación Marqués de Valdecilla), Santander, Cantabria Spain; 4 Universidad de Cantabria, Santander, Spain; 5 CIBER Epidemiología y Salud Pública (CIBERESP), Madrid, Spain

**Keywords:** Osteoporosis, Sarcopenia, Obesity, Sarcopenic osteoporosis, Sarcopenic obesity

## Abstract

**Summary:**

The associations of sarcopenia with osteoporosis or obesity have a very low prevalence. No trend towards an association between osteoporosis and sarcopenia is observed. Sarcopenia and obesity tend not to coincide, as if they were antagonistic disorders.

**Purpose:**

To know (a) the prevalence in our region of sarcopenic osteoporosis (association of sarcopenia and osteoporosis (T-score < − 2.5)), sarcopenic obesity, and the association of osteoporosis, sarcopenia, and obesity; (b) the tendency of osteoporosis, sarcopenia, and obesity to associate with each other; and (c) the bone mineral density (BMD), the components of sarcopenia, and the prevalence of fragility fractures in these associations.

**Methods:**

The study was performed in the Camargo cohort. Osteoporosis was diagnosed by DXA, sarcopenia by the EWGSOP-1 criteria, and obesity by body mass index (BMI) and fat percentage. Fractures were verified radiographically or by consulting the medical records.

**Results:**

The prevalence of sarcopenic osteoporosis was 2.8% and the OR for this association 1.03 (*p* = 0.89). The prevalence of sarcopenic obesity by BMI was 1.4% and by fat percentage 5.9% (corresponding ORs: 0.18 (p < 0.0001) and 0.58 (*p* < 0.003) respectively). The prevalence of the association of osteoporosis, sarcopenia, and obesity was 0.0% when assessed by BMI and 0.8% when assessed by fat percentage. Patients with sarcopenic osteoporosis have less muscle mass and more fragility fractures than sarcopenic patients overall. In patients with sarcopenic obesity by fat percentage, muscle mass and strength, as well as physical performance, were similar to those of sarcopenic patients overall. Neither BMD nor fracture prevalence showed differences between patients with sarcopenic obesity and patients with sarcopenia or obesity in general.

**Conclusion:**

Our study supports the idea that the prevalence of the mixed disorders studied is low. No significant association between osteoporosis and sarcopenia was found. Sarcopenia and obesity seem to tend to occur in different people, as if suffering from one of them hinders suffering from the other.

## Introduction

Increasing life expectancy is leading to a progressive aging of the population [1], which translates into an increase in the prevalence of diseases and disorders associated with old age. Some of these disorders may coincide merely because they occur mainly at this stage of life, but others may coincide also because they have common pathophysiological mechanisms, or even because they are reciprocally co-determined.

One of the conditions associated with aging is sarcopenia [2]. Described just over three decades ago [3] as a loss in muscle mass, a decrease in muscle strength and functional performance have subsequently been added as characteristic components of this disease [4]. Impaired muscle function may predispose to falls [5], and falls can be followed by fractures, which connects sarcopenia with osteoporosis.

Osteoporosis is another process associated with old age that predisposes to fractures [6]. Sarcopenia and osteoporosis may coincide because both are associated with aging, but also because they may have common etiological factors (i.e., physical inactivity) [7] and even because muscular alterations may have repercussions on bone and vice versa [8]. This has led to consider the existence of a mixed syndrome that encompasses the two processes. Some authors have suggested the term “osteosarcopenia” [9, 10] to designate a disorder characterized by the coexistence of sarcopenia and bone mass with a T-score < − 1.0 (osteopenia and osteoporosis). Such a term excludes a situation that we believe it is important to consider: the association of sarcopenia and osteoporosis alone (T-score < − 2.5). In this study, we will focus on this association, which we will refer to as “sarcopenic osteoporosis.”

Furthermore, in recent years, there has been growing concern about the possible association of sarcopenia with obesity, as aging is associated with a decrease in muscle mass and an increase in fat mass [11]. This has led to coin the term “sarcopenic obesity” [12]. Adiposity can have a detrimental effect on muscle by infiltrating it with fat [13]. The alteration of muscle function, in turn, can facilitate falls and ultimately increase—at least theoretically—the risk of fractures.

If sarcopenia may tend to be associated with osteoporosis on the one hand and obesity on the other, it is conceivable that there is also a tendency for all three conditions to be associated. The term “osteosarcopenic obesity” [14] has been coined to name the association of obesity, sarcopenia, and osteopenia/osteoporosis. As we are interested in the study of the association of obesity, sarcopenia, and osteoporosis (T < − 2.5), we will refer to this association as “sarcopenic osteoporotic obesity.”

The study of the epidemiology of the aforementioned disorders is hampered by the fact that there is no single universally agreed definition for any of them. Osteoporosis is usually defined densitometrically, according to the WHO definition (T-score ≤ − 2.5) [15, 16], but there are discrepancies as to whether the measurement should be made only at the femoral neck (FC) [17], or can also be made at the lumbar spine (LS) or total hip (TC) [18]. Obesity can be diagnosed according to anthropometric criteria (BMI) [19] or according to the percentage of body fat (for which, in turn, several thresholds have been proposed [20]). Finally, there is a large number of definitions of sarcopenia [4], which for reasons of space we will not consider here. The most widely used is that of the European Working Group on Sarcopenia in Older People (EWGSOP) [21], which has recently been modified [22] (now being referred to as EWGSOP-1 and EWGSOP-2 respectively).

If, due to the multiplicity of definitions, there are difficulties in studying the epidemiology of each of these three disorders separately, the study of the mixed disorders should understandably be greater. Although we are aware of the limitations that this entails, the absence of studies on these disorders in our region has prompted us to address this subject. The criteria used will be clearly stated, so that the results can be compared in the best possible way with those of other similar studies. In addition to addressing the epidemiological data, we will attempt to characterize these disorders in terms of their bone and muscle features.

## Subjects and methods

### Subjects

The study was carried out with people included in the Camargo cohort, a prospective community-based cohort designed to evaluate the prevalence and incidence of metabolic bone diseases and risk factors for osteoporosis and fragility fractures. The cohort was set up with postmenopausal women and men aged 50 years and older attending a Primary Care Center in Northern Spain for medical reasons or for their regular health examination. Details of the Camargo Cohort Study have been previously reported [23, 24]. Clinical data and biochemical parameters were assessed also as previously published [23, 24].

Camargo cohort participants are monitored every 4 years. In the third survey, data were also collected to assess the presence of sarcopenia. At this time, the fat percentage was also determined. Although the total cohort consisted of 3000 participants, for the present study, only the first 1000 people who were studied in this third evaluation were included. The study protocol was approved by the “Comité Ético de Investigación Clínica de Cantabria-IDIVAL,” internal code 2016.003, and all patients gave written informed consent.

### Sarcopenia assessment

Sarcopenia was diagnosed when patients had low muscle mass plus one of the two following conditions: low muscle strength or low physical performance (EWGSOP-1). The modification of EWGSOP-2 was published after we started our study, and we kept the first definition. Muscle mass was defined as appendicular muscle mass kilogram per square meter. Appendicular muscle mass was measured from a total body scan performed at the same time as the BMD assessment using a DXA densitometer (Hologic QDR 4500, Waltham, MA, USA). Cutoff points to diagnose low muscle mass were 5.5 kg/m^2^ for women and 7.26 kg/m^2^ for men. Muscle strength was assessed as handgrip strength using a handgrip dynamometer (Jamar 5030). Low handgrip strength was defined as ≤ 30 kg for men and ≤ 20 kg for women. Physical performance was evaluated using the gait speed test, by means of measuring the time in seconds required to cover a distance of four meters at the normal walking speed (m/s). Low gait speed was defined as ≤ 0.8 m/s. The occurrence of a low muscle mass with normal muscle strength or physical performance was diagnosed as “presarcopenia.”

### Bone densitometry

BMD was measured by DXA at the LS including L1-L4, FN, and TH. In vivo precision was 0.4–1.5% at the different measurement sites. Results were expressed as gr/cm^2^. Osteoporosis was considered to be present when T-score was ≤ − 2.5 at the LS, FN, or TH. A T-score between − 1.0 and − 2.5 qualified as osteopenic. Reference values were derived from the NHANES III normative data for hip sites and from the manufacturer’s reference range for the lumbar spine.

### Identification of vertebral and non-vertebral fractures

Vertebral fractures were assessed using spinal lateral X-rays. Vertebral fractures were evaluated according to a semiquantitative approach [25]. Radiographs were examined independently by two of the authors, blinded to any other clinical data of the participants. Disagreements were resolved by consensus. Non-vertebral fractures unrelated to major trauma were self-reported and later confirmed by examination of medical or radiological reports.

### Sarcopenic osteoporosis

“Sarcopenic osteoporosis” was defined as the association of osteoporosis (T score ≤ − 2.5) and sarcopenia. We purposely distinguish it from the term “osteosarcopenia,” which is being used mostly to refer to the association of sarcopenia with osteoporosis or osteopenia (T-score ≤ − 1.0).

### Sarcopenic obesity

We have considered obesity in two ways: first, as a BMI greater than 30 kg/m^2^ and, second, as a percentage of fat greater than 40% in women and 28% in men [26]. Fat percentage was determined by DXA.

### Sarcopenic osteoporotic obesity

As the name suggests, by osteoporotic sarcopenic obesity, we mean the coexistence of obesity, sarcopenia, and osteoporosis, as defined previously.

### Statistical analysis

Results are expressed as mean ± standard deviation (SD) or percentages, as appropriate*.* Differences between groups were evaluated using the *t*-Student test or the chi-squared test for continuous and categorical variables, respectively. Logistic regression models (adjusted and not adjusted) were used to determine the association between the different conditions and the results are shown in terms of odds ratio (OR) with 95% confidence intervals (95% CIs). Correlations between two sets of data were calculated using Pearson’s correlation coefficients.

All analyses were conducted using SPSS 15.0 (Chicago, IL, USA) and STATA 16/SE (Stata Corp., College Station, TX, USA). The level of significance was set at *p* < 0.05.

## Results

We studied 1000 people (751 females, 249 males), whose general characteristics are shown in Table 1. Table 2 shows the values of the diagnostic components of sarcopenia (muscle mass, muscle strength, physical performance) and those of BMD, as well as the prevalence of fractures, for the whole population and for people with sarcopenia, osteoporosis, sarcopenic osteoporosis, obesity, sarcopenic obesity, and sarcopenic osteoporotic obesity (obesity diagnosed by fat percentage).

**Table 1:** Characteristics of participants

Age (years)	71.94 ± 7.53
Sex (women %)	75.1%
Weight (kg)	72.46 ± 17.02
Height (cm)	155.92 ± 15.00
BMI (kg/m^2^)	28.50 ± 4.73

**Table 2:** Muscle components of sarcopenia, BMD, and fractures

	Whole population	Sarcopenic subjects	Osteoporotic subjects	Subjects with sarcopenic osteoporosis	Obesity by % fat	Sarcopenic obesity by % fat	Sarcopenic osteoporotic obesity by % fat
	*n* = 1000	*n* = 141	*n* = 204	*n* = 26	*n* = 557	*n* = 59	*n* = 8
Muscle mass (kg/m^2^)	7.08 ± 1.34^a^	6.05 ± 1.00^ g^	6.47 ± 0.99 ^j^	5.36 ± 0.52^o^	7.20 ± 1.53^q^	6.20 ± 1.06^ s^	5.36 ± 0.77
Muscle strength (kg)	23.17 ± 8.81^b^	18.73 ± 5.21^ h^	20.1 ± 7.0^ k^	17.82 ± 3.62^p^	22.83 ± 9.0^r^	18.58 ± 5.42^t^	17.45 ± 4.21
Physical performance (m/s)	1.13 ± 1.37^c^	1.02 ± 0.33^i^	1.09 ± 0.32	1.01 ± 0.19	1.14 ± 1.41^e^	1.07 ± 0.37	1.08 ± 0.20
BMD-LS (g/cm^2^)	0.914 ± 0.166^d^	0.920 ± 0.148	0.850 ± 0.166^ l^	0.887 ± 0.133	0.918 ± 0.173	0.892 ± 0.172	0.835 ± 0.200
BMD-FN (g/cm^2^)	0.745 ± 0.117^e^	0.739 ± 0.106	0.709 ± 0.121^ m^	0.717 ± 0.103	0.756 ± 0.115	0.738 ± 0.107	0.699 ± 0.097
BMD-TH (g/cm^2^)	0.875 ± 0.144^f^	0.867 ± 0.118	0.830 ± 0.134^n^	0.834 ± 0.117	0.890 ± 0.136	0.879 ± 0.124	0.846 ± 0.819
Fractures	16.4%	20.6%	27.5%	35.7%	15.9%	15.3%	25%

*BMD*, bone mineral density; *LS*, lumbar spine; *FN*, femoral neck; *TH*, total hip

Crude *p* values (“t” test): *p* ≤ 0.0001: a versus g; b versus h, c versus i; a versus j; b versus k; d versus l; e versus m; f versus n; g versus o; h versus p; j versus o; g versus s; *p* = 0.0004: r versus t. Adjusted (sex and age) *p* values are given in the text

### Sarcopenic osteoporosis

1. Sarcopenia, osteoporosis, and fragility fractures
  The prevalence of sarcopenia was 14.1% and that of presarcopenia 40.3%. The mean age of the sarcopenic patients was 72.98 ± 8.16 years. In sarcopenic patients, muscle mass, muscle strength, and physical performance were 15%, 20%, and 10% lower than in the general population, respectively (*p* < 0.0001 in all cases) (Table 2). After adjusting for age and sex, the difference in physical performance was no longer significant. The BMD of sarcopenic patients did not differ from that of the overall population.
  The prevalence of osteoporosis was 20.4%. The mean age of the osteoporotic patients was 72.14 ± 8.09 years. In osteoporotic patients, BMD at the lumbar spine, femoral neck, and total hip was 7% (*p* < 0.0001), 5% (*p* < 0.0001), and 5% (*p* = 0.0001) lower than in the general population, respectively (Table 2). Muscle mass and strength, but not physical performance, were significantly lower (*p* < 0.0001) in patients with osteoporosis than in the whole population. After adjusting for age and sex, however, the difference in muscle strength was no longer significant.
  The percentage of people with fragility fractures in the total population was 16.4%. As expected, the percentage was higher in patients with osteoporosis (27.5%; *p* = 0.0002). In patients with sarcopenia (20.6%), the difference was not significant either compared to the total population or to patients with osteoporosis without sarcopenia.
2. Association of sarcopenia and osteoporosis: sarcopenic osteoporosis

2.1 Prevalence of sarcopenic osteoporosis and odds ratio (OR) for the association between sarcopenia and osteoporosis

The prevalence of sarcopenic osteoporosis in the general population was only 2.8%. The mean age of the patients with this disorder was 69.96 (± 6.75) years, no significantly different from that of the sarcopenic or osteoporotic patients. Sarcopenia was present in 13.7% of osteoporotic people and 14.2% of non-osteoporotic people. On the other hand, osteoporosis was present in 19.9% of sarcopenic patients and 20.5% of non-sarcopenic people. The sex and age adjusted OR was 1.03 (0.66–1.62; *p* = 0.89). Therefore, a tendency towards an association between sarcopenia and osteoporosis diagnosed with the criteria stated above was not observed.

In spite of these OR values, muscle mass and muscle strength were lower in people with vs. without osteoporosis (*p* < 0.0001 in both cases) (Table 2). The difference, however, was no longer significant for muscle strength after adjusting for age and sex. A significant, albeit small, relationship was observed between muscle mass and BMD at the femoral neck and total hip (*r* = 0.12 and *r* = 0.14 respectively; *p* < 0.01 in both cases).

2.2 Sarcopenic components and BMD in patients with sarcopenic osteoporosis vs. those with only sarcopenia or osteoporosis

Muscle mass and muscle strength were lower in patients with sarcopenic osteoporosis than in patients with sarcopenia in general (*p* < 0.0001 in both cases, although after adjusting for age and sex, significance was lost for muscle strength). In contrast, BMD did not differ between patients with sarcopenic osteoporosis and osteoporotic patients overall (Table 2).

2.3 Fragility fractures in patients with sarcopenic osteoporosis

The prevalence of fragility fractures in patients with sarcopenic osteoporosis was 35.7%, slightly more than double that in the global population (16.4%) and also higher than in patients with sarcopenia without osteoporosis (16.8%) (*p* = 0.0095 and *p* = 0,003, respectively, after adjusting for age and sex). Although the percentage was also higher than that of patients with osteoporosis without sarcopenia (26.1%), the difference was not statistically significant (*p* = 0.42).

### Sarcopenic obesity

1. Obesity diagnosed by BMI
  The percentage of patients with a BMI > 30 k/m^2^ in the whole population was 34.2%, but the number of people who presented simultaneously with obesity and sarcopenia (sarcopenic obesity) was only 14, which means 1.4% of the total population, and 9.9% of people with sarcopenia. The percentage of patients with obesity among non-sarcopenic people was 38.2%. The percentages of patients with sarcopenia among obese and non-obese people were 4.0% and 19.3%, respectively. The OR, adjusted for age and sex, was 0.18 (0.10–0.31) (*p* < 0.0001). Given the small number of patients with sarcopenic obesity diagnosed by BMI, no further statistical analysis were performed. Only one person out of the 14 with sarcopenic obesity diagnosed by BMI had a fragility fracture.
2. Obesity diagnosed by the percentage of body fat
  The prevalence of obesity diagnosed according to body fat percentage was 52.7%, higher than that observed for people with a BMI > 30 kg/m^2^ (*p* < 0.0001). The number of people who simultaneously presented obesity and sarcopenia was 59, i.e., 5.9% of the total population (compared to 1.4% when obesity was diagnosed by BMI; *p* < 0.0001) and 42% of people with sarcopenia (much higher than the 9.9% of people diagnosed with obesity by the anthropometric criteria; *p* < 0.0001). Thus, the prevalence of obesity in the population as a whole is 1.5 times higher when diagnosed with the fat percentage criterion, but the prevalence of sarcopenic obesity is even higher: 4.2 times. The percentage of people without sarcopenia that were obese by the fat percentage criterion was 54.5%. On the other hand, 11.2% of obese people and 17.3% of non-obese people were sarcopenic. The OR adjusted for age and sex between sarcopenia and obesity diagnosed by this criterion was 0.58 (0.41–0.86) (*p* < 0.003).
  As expected, muscle mass and strength were significantly lower in patients with sarcopenic obesity than in obese people overall (*p* < 0.0001 and < 0.0004 respectively); however, they were similar to those of patients with sarcopenia as a whole (Table 2). Physical performance was not significantly different from that of obese or sarcopenic patients.
  Interestingly, when obesity is diagnosed by fat percentage, muscle mass is lower than when obesity is diagnosed by BMI (7.20 ± 1.53 vs. 7.81 ± 1.57 kg/m^2^; *p* < 0.0001). In patients with sarcopenic obesity, muscle mass is also lower when obesity is diagnosed by fat percentage than by BMI, but the difference is smaller and not significant (6.20 ± 1.06 vs. 6.43 ± 0.87 kg/m^2^; *p* = 0.45). The small sample sizes (*n* = 59 and 14, respectively) may contribute to this (data for obesity and sarcopenic obesity diagnosed by BMI are not shown in Table 2).

### Sarcopenia, obesity, and osteoporosis (“sarcopenic osteoporotic obesity”)

1. Obesity diagnosed by BMI
  There were no individuals who associated sarcopenia, osteoporosis, and obesity diagnosed by this criterion. That is to say: no person had what we have called “sarcopenic osteoporotic obesity” when obesity was diagnosed according to BMI.
2. Obesity diagnosed by the percentage of body fat
  When obesity was diagnosed by the fat percentage criteria, 8 patients had simultaneous obesity, sarcopenia, and osteoporosis. This accounts only for 0.8% of the total population. The OR for the association of obesity with sarcopenic osteoporosis, adjusted for age and sex, was 0.38 (0.16–0.89; *p* = 0.026), and the adjusted OR for the association of osteoporosis with sarcopenic obesity was 0.65 (0.30–1.41, *p* = 0.28). Given the small number of patients with this disorder, no further statistical analysis were performed, although their muscle component and BMD values are given in Table 2. The overall impression is that the muscle components of sarcopenia and BMD show a similar pattern to that observed in patients with sarcopenic osteoporosis in general.

## Discussion

### Sarcopenic osteoporosis

In the population studied by us (mean age 72 years), with the criteria used to diagnose sarcopenia and osteoporosis, the prevalence of sarcopenia was 14.1%, that of osteoporosis 20.4% and that of sarcopenic osteoporosis 2.8%.

The prevalence of sarcopenia reported by others is highly variable, depending on the criteria used for its diagnosis, the techniques used to measure its components, and the type of people studied. In a population with similar characteristics to ours, the estimated prevalence of sarcopenia ranged from 8.4 to 27.6%, according to the method of diagnosis. The prevalence of sarcopenia assessed with the EWGSOP-1 criterion in five papers collected in a systematic review [27] was 4.3%, 5.6%, 11.2%, 15.9%, and 31.9%. Mayhew et al. [28] found that with the EWGSOP/AWGS definitions, the pooled prevalence estimates was 12.9% (9.9–15.9%). Locquet et al. [29] with criteria similar to ours found a similar prevalence as well (14.9%). From all the above, we could draw two conclusions: first, there is a great variability in the figures reported and, second, the prevailing values with the EWGSOP-1 criterion seem to be between 10 and 15%, figures which are consistent with our data.

The prevalence of sarcopenic osteoporosis (sarcopenia plus T-score < − 2.5 T) has been little studied. Loquet et al. [29] found figures not very far from ours (4.5%). However, most authors have published the prevalence of the combination of sarcopenia and T-Score < − 1.0 (“osteosarcopenia”), understandably reporting higher (although very variable) values. A recent review [30] has reported figures ranging from 5 to 37%. For comparative purposes, we have assessed the prevalence of the combination of sarcopenia plus T-score < − 1.0 in our cohort, the figure being 10.5%.

We have found no significant association between sarcopenia and osteoporosis, contrary to others [31, 32]. Of note, however, although we found no association between the two diseases (which are, by definition, binary variables: yes/no), we found a slight relationship between the continuous variables that underlie the diagnosis of these entities (i.e., muscle mass and hip BMD). Other studies have found similar relationships between low muscle mass and bone mass [33], as well as between a decline in muscle strength and a decrease in spine BMD [34].

The prevalence of fractures in patients with sarcopenic osteoporosis was 35.7%, significantly higher than in patients with sarcopenia without osteoporosis (16.8%; *p* = 0.0095), but not that of patients with osteoporosis without sarcopenia (26.1%; *p* = 0.45). Nor did we find significant differences between the prevalence of fractures in patients with sarcopenia (20.6%) and patients without it (15.7%) in the population as a whole. (figures for patients with sarcopenia without osteoporosis, osteoporosis without sarcopenia, or the general population without sarcopenia are not shown in Table 2). Therefore, our data do not indicate that sarcopenia increases the risk of fractures and suggest that the rise in fractures in patients with sarcopenic osteoporosis is mainly driven by osteoporosis. In fact, a review [27] of the studies carried out on this topic indicates that the impact of sarcopenia on fractures development is controversial since data have been published in both directions. Beaudart et al. [35] also point out that the evidence for a link between sarcopenia and fracture is not consistent.

### Sarcopenic obesity

In our population, the prevalence of obesity diagnosed by BMI was 34.2%, while by the percentage of fat it was 52.7%. The prevalence of sarcopenic obesity was 1.4% when obesity was diagnosed by BMI and 5.9% when it was diagnosed by the percentage of body fat (4.2 times higher). The fact that the sarcopenic obesity prevalence is higher when obesity is diagnosed by fat percentage must be attributed to the fact that the calculation of the BMI takes into account the weight of the body (weight/m^2^), which is influenced by muscle mass. The prevalence of sarcopenic obesity that has been reported in other studies is highly variable. A German study [36] in women over 70 years using the EWGSOP-1 definition of sarcopenia found a prevalence of sarcopenic obesity of 0% when obesity was diagnosed by BMI and of 2.3% with the percentage of fat criterion. A recent Indian study [37] applying the EWGSOP-2 definition of sarcopenia has reported a prevalence of sarcopenic obesity in the over-65 population of 3.8% in women and 6.7% in men when obesity was diagnosed by BMI, and 5.7% in women and 6.7% in men when diagnosed by fat percentage. The data reported in both studies are fairly similar to ours. However, those published by other authors are much higher. For instance, a German study [38] in men over 65 years of age, with the EWGSOP criteria for sarcopenia and BMI for obesity has reported a prevalence of 15.6%. A Lebanese study [39] carried out in people over 60 years of age, assessing sarcopenia by BMI-adjusted appendicular lean mass and obesity by fat percentage found prevalence figures of 33.5% in women and 12.5% in men.

In the present study, the OR for the association “sarcopenia”- “obesity by BMI” was 0.18 (*p* < 0.0001) and for the association “sarcopenia”- “obesity diagnosed by body fat percentage” 0.58 (*p* = 0.003). It is understandable that the OR is lower when obesity is diagnosed on the basis of weight (BMI = weight/m^2^), for the reason already stated that weight is influenced by muscle mass. Hence, to the extent that weight contributes to a high BMI, this form of obesity is less likely to be associated with sarcopenia. In any case, it seems that sarcopenia and obesity tend not to coincide, as if the presence of one of them reduces the chances of suffering from the other. It has recently been emphasized that obesity can have a deleterious effect on muscle, as it leads to its infiltration by fatty tissue, which in turn produces adipokines that are detrimental to muscle fibers. The relationship between adipose tissue and muscle, however, is more complex than that brought about by its fatty infiltration. For example, contrary to the possible tendency of obesity to be associated with sarcopenia, it has been stated that increments in skeletal muscle mass may occur in obesity as a consequence of the fact that excess weight places a greater mechanical load on muscles for any type of movement or exercise to be performed. In addition, the excessive caloric intake that has led to obesity may have been accompanied by a good protein intake. Therefore, different fat-muscle relationships can probably be distinguished depending on the factors involved.

Regarding bone health, BMD measurements in sarcopenic obesity in our cohort—whatever the criteria used to diagnose obesity—were no different from those of patients with obesity or sarcopenia alone nor from those of the overall population. Muscle components, as expected, showed or tended to show lower values than those of obese people without sarcopenia.

### Sarcopenic osteoporotic obesity

No case of association between sarcopenia, obesity, and osteoporosis (T-score < − 2.5) was observed in our study population when obesity was diagnosed by BMI. When diagnosed by fat percentage, eight individuals were identified in whom all three disorders coincided. This represents a very low prevalence in the population as a whole (0.8%). We are not aware of studies that have addressed this association. However, data have been published on the association of obesity, sarcopenia, and BMD with a T-score < − 1.0. As expected, the values are higher, and also very variable, depending on the definition criteria used and the population studied. They range from 4.1 [40] to 19% [41], with the most frequent values reported being around 10–12% [42–44]. For the sake of comparison, and since the figure of 0.8% observed in our study refers to the association of obesity, sarcopenia, and osteoporosis sensu stricto (T-score < − 2.5), we have also calculated the prevalence in people with obesity, sarcopenia, and T-score < − 1.0 in the people studied by us. The figure found was 4.0%.

The OR for the association of obesity with sarcopenic osteoporosis was low (0.38; *p* = 0.026). Accordingly, it can be concluded that, similar to what we have discussed regarding the association of sarcopenia with obesity, sarcopenic osteoporosis does not tend to present simultaneously obesity, but rather the opposite: it tends not to do so.

In the eight patients in whom obesity, sarcopenia, and osteoporosis (T-score < − 2.5) occurred together in our study, the muscular components of sarcopenia and BMD showed a pattern similar to that observed in patients with sarcopenic osteoporosis. Therefore, it seems that, in this regard, obesity does not add much to sarcopenic osteoporosis. Pang et al. [45] have also not found that the coincidence of the three diseases is accompanied by a functional impairment greater than that of sarcopenia or sarcopenic obesity.

### General comments and conclusion

There are pathophysiological reasons for sarcopenia and osteoporosis to be associated and even for the association of sarcopenia and obesity. It has even been pointed out that there are reasons to believe that obesity and osteoporosis may also be associated. Consequently, it is conceivable that the three disorders may be combined. However, whether such associations may be regarded as new clinical entities is controversial. One of the reasons why more decisive conclusions have not yet been reached is the fact that there are different definitions for each of the three disorders (something particularly striking in the case of sarcopenia). It is possible that, with some definitions, the associations (or at least some of them) may indeed be considered as new clinical entities, while with others they may not. Clearly, further studies are required to clarify these issues. In this context, we feel it is worth noting the scarcity of studies performed regarding the association of sarcopenia or obesity with osteoporosis sensu stricto (T < − 2.5). Thereupon, we believe that our study represents an important contribution in this field.

Our study has some limitations. First, it has been done with the EWGSOP-1 definition and not with the revised EWGSOP-2. We considered starting again with the new definition, but we thought that ending the study with the EWGSOP-1 criteria had the advantage of allowing us to compare our results with a larger number of studies since most of the research on this subject published up to that time was carried out with these criteria. Second, our study has the limitations inherent to cross-sectional studies. Therefore, causal relationships cannot be established. Third, it has been carried out on people from a specific region of northern Spain, so its results may not be extrapolable to other parts of the world or even other regions of our country. Moreover, the sample was compounded by functionally independent community-dwelling subjects, and therefore, the results may not apply to more frail people. However, these issues do not decrease the interest of our results since the main objective of our study was to know the epidemiological data of the associations studied in community-dwelling people from our region.

**Fig. 1:**
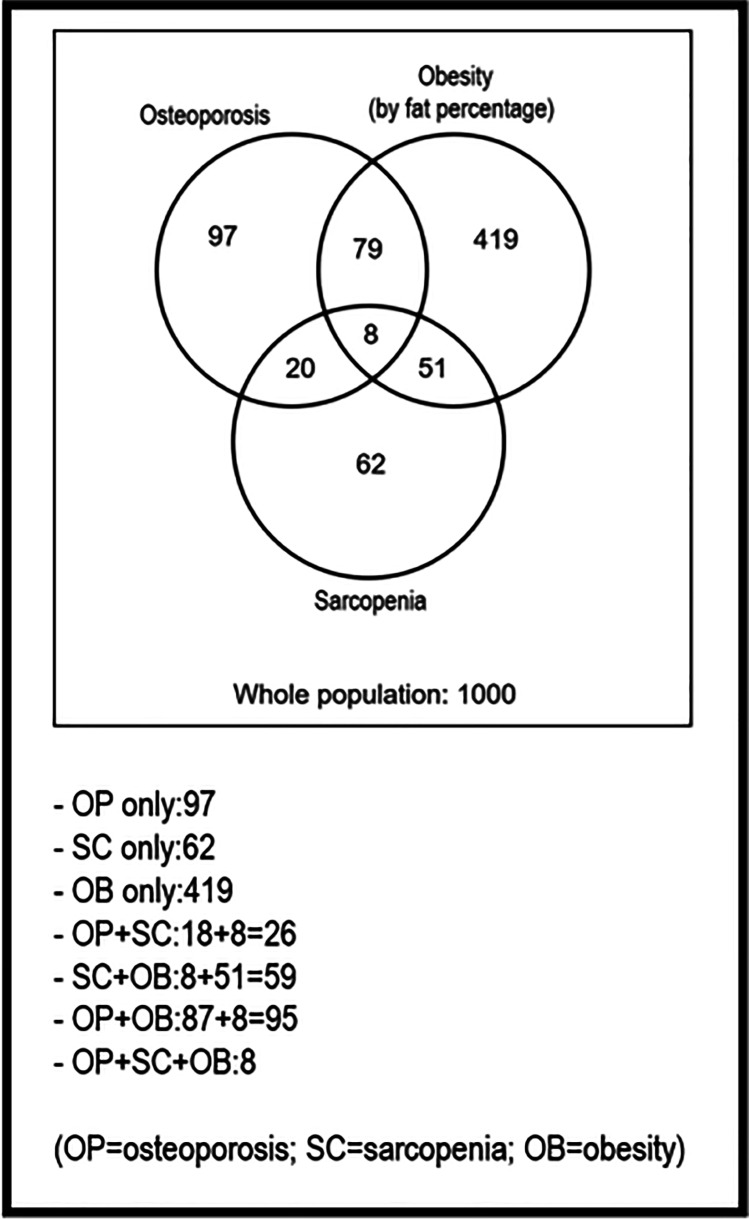
Venn diagram showing the absolute number of patients in which two of the three disorders considered, or all three together, coincide

Our study was based on a large sample (1000 people). Nevertheless, the low prevalence of the associations studied (2.8% sarcopenic osteoporosis; 5.9% sarcopenic obesity; and 0.8% sarcopenic osteoporotic obesity—defining obesity as percentage of fat) makes the sample size of these associations too small to be able to study functional aspects in depth (Fig. 1 provides the absolute figures). Hence, it is necessary to start from larger population samples to be able to characterize these subpopulations in detail.

To conclude, we have found in our study that the prevalence of sarcopenic osteoporosis is very uncommon, with no tendency for sarcopenia and osteoporosis to be associated. Besides, sarcopenia does not seem to increase the tendency to fractures. The prevalence of sarcopenic obesity is also very rare, and the frequency of obesity in sarcopenic patients is lower than in the general population. Moreover, the concurrence of obesity is not accompanied by further muscle impairment. The coexistence of sarcopenia, osteoporosis, and obesity in community-dwelling persons is enterally exceptional.
